# Intimal CD31-Positive Relative Surfaces Are Associated with Systemic Inflammatory Markers and Maturation of Arteriovenous Fistula in Dialysis Patients

**DOI:** 10.3390/jcm12134419

**Published:** 2023-06-30

**Authors:** Réka Kaller, Eliza Russu, Emil Marian Arbănași, Adrian Vasile Mureșan, Márk Jakab, Claudiu Constantin Ciucanu, Eliza Mihaela Arbănași, Bogdan Andrei Suciu, Ioan Hosu, Liliana Demian, Emőke Horváth

**Affiliations:** 1 Clinic of Vascular Surgery, Mures County Emergency Hospital, 540136 Targu Mures, Romaniaemil.arbanasi@umfst.ro (E.M.A.); claudio.ciucanu@gmail.com (C.C.C.); 2 Doctoral School of Medicine and Pharmacy, George Emil Palade University of Medicine, Pharmacy, Science and Technology of Targu Mures, 540139 Targu Mures, Romania; 3 Department of Vascular Surgery, George Emil Palade University of Medicine, Pharmacy, Science and Technology of Targu Mures, 540139 Targu Mures, Romania; 4 Faculty of Medicine, George Emil Palade University of Medicine, Pharmacy, Science and Technology of Targu Mures, 540139 Targu Mures, Romania; mark.jakab@icloud.com; 5 Faculty of Pharmacy, George Emil Palade University of Medicine, Pharmacy, Science and Technology of Targu Mures, 540139 Targu Mures, Romania; arbanasi.eliza@gmail.com; 6 Department of Anatomy, George Emil Palade University of Medicine, Pharmacy, Science and Technology of Targu Mures, 540139 Targu Mures, Romania; bogdan.suciu@umfst.ro; 7 Department of Nephrology, Mures County Emergency Hospital, 540136 Targu Mures, Romania; 8 Center of Advanced Medical and Pharmaceutical Research, George Emil Palade University of Medicine, Pharmacy, Science and Technology of Targu Mures, 540139 Targu Mures, Romania; lilidemian@yahoo.com; 9 Department of Pathology, George Emil Palade University of Medicine, Pharmacy, Science and Technology of Targu Mures, 540139 Targu Mures, Romania; emoke.horvath@umfst.ro

**Keywords:** arteriovenous fistula, AVF, CD31-positive relative surface, IL-6, inflammation, dialysis, end-stage kidney disease

## Abstract

Background: Arteriovenous fistula dysfunction is a widely disputed subject in the scientific literature on end-stage kidney disease (ESKD). The main cause of mortality and morbidity in these patients is the non-maturation or dysfunction of the arteriovenous fistula. Despite the many complications, the native arteriovenous fistula remains the gold standard in the treatment of these patients requiring renal replacement. This study aims to discuss the predictive role of some systemic inflammatory biomarkers (NLR, PLR, SII, IL-6), intimal hyperplasia, and neoangiogenesis (characterized by intimal-media CD31-positive relative surface) in arteriovenous fistula maturation failure. Methods: The present study was designed as an observational, analytical, and prospective study which included patients diagnosed with ESKD with indications of radio-cephalic arteriovenous fistula (RCAVF). Demographic data, comorbidities, preoperative laboratory data and histological/digital morphometry analysis results were processed. The patients included were divided into two groups based on their AVF maturation status at 8 weeks: “Maturation” (Group 1) and “Failed Maturation” (Group 2). Results: There was no difference in the demographic data. In terms of comorbidities, the second group had a greater incidence of heart failure (*p* = 0.03), diabetes (*p* = 0.04), peripheral artery disease (*p* = 0.002), and obesity (*p* = 0.01). Additionally, regarding the laboratory findings, these patients had higher levels of serum uric acid (*p* = 0.0005), phosphates (*p* < 0.0001), and creatinine (*p* = 0.02), as well as lower levels of total calcium (*p* = 0.0002), monocytes (*p* = 0.008), and lymphocytes (*p* < 0.0001). Moreover, all inflammatory markers (*p* = 0.001; *p* < 0.0001; *p* = 0.006, and *p* = 0.03) and Ca-P product (*p* < 0.0001) had higher baseline values in Group 2. Upon immunohistochemical analysis, regarding the density of neoformed vessels, there was a higher incidence of CD31-positive surfaces (*p* = 0.006) and CD31-positive relative surfaces (*p* = 0.001); the NLR (r = 0.323; *p* = 0.03), PLR (r = 0.381; *p* = 0.04), SII (r = 0.376; *p* = 0.03), and IL-6 (r = 0.611; *p* < 0.001) are all significantly correlated with vascular density, as evidenced by CD31. Conclusions: Heart failure, peripheral artery disease, obesity, and diabetes, as well as the systemic inflammatory markers (NLR, PLR, SII, IL-6), intimal hyperplasia, and CD31-positive relative surfaces are predictors of arteriovenous fistula maturation failures.

## 1. Introduction

Non-maturation of the arteriovenous fistula or its dysfunction remains the leading cause of morbidity and mortality in patients with stage 5 chronic kidney disease (CKD). Despite that, the Cimino fistula remains the gold standard in the treatment of ESKD in comparison with catheters and grafts [1].

The current literature suggests that the arteriovenous fistula (AVF) is the optimal vascular access route for dialysis in patients with end-stage kidney disease (ESKD) [2,3], with patency rates at one year ranging from 52–71% for the radiocephalic AVF (RCAVF) [4,5,6,7,8,9,10,11], 75–89% for the brachiocephalic AVF (BCAVF) [12,13,14,15,16,17,18], and 64–89% for the brachiobasilic AVF [12,16,19,20,21,22,23]. According to the above-mentioned publications, early failure occurs in between 5 and 37% of patients in the case of RCAVF (4–11), 8–16% in the case of BCAVF [12,13,14,15,16,17,18], and 2–23% in the case of BBAVF, all within one month after access creation [12,16,19,20,21,22,23]. 

Significant arterial atherosclerotic damage, juxta-anastomotic stenosis of the venous segment, or the presence of intimal hyperplasia (IH) with or without penetrating capillaries within neointima-media at the venous segment level, which limits the appropriate dilation and development, are the most frequent causes of failure of AVF maturation [24,25,26]. Wali et al. [27,28] were the first to draw attention to the presence of IH in patients with chronic renal disease. More recently, numerous publications have analyzed maximal intimal thickness [29,30,31,32,33] and its associations with AVF outcomes from a morphometric standpoint, but with inconsistent results. Some mechanical factors can also influence the success of the procedure before the fistula is performed. Preoperative punction of the vein, long-term hospitalization of the patient with intravenous treatment, and dehydration can have a negative impact on the quality of the vein, and can lead to venous intimal hyperplasia. 

In comparison to the results reported by Tabbara et al. [29] (*p* = 0.2), Martinez et al. [30] (*p* = 0.70), and Allon et al. [31] (*p* = 0.49), only Lee et al. [26] revealed that the presence of neo-intimal hyperplasia is associated with maturation failure (*p* = 0.03). Furthermore, the mechanism through which CKD influences the development of IH is not fully understood. Some immunohistochemical studies [26,32,34,35] have identified a low number of CD3+ or CD68+ inflammatory cells in the venous wall. Wasse et al. [34] also discovered the presence of interleukin-6 (IL-6) and tumor necrosis factor-alpha (TNF-α) expression. IL-6 and TNF-α were initially shown to have cytotoxic effects on the endothelium, causing inflammation [36]. The primary goals of this study are to examine the association between the systemic inflammatory state of patients and intimal thickness and its degree of neovascularization at the venous segment level of AVF, assessing them as possible risk factors for AVF maturation failure.

## 2. Materials and Methods

### 2.1. Study Design

The current study included 42 patients with ESRD, hospitalized at the Vascular Surgery Clinic of Targu Mures Emergency County Hospital in Romania from January 2020 to December 2021, with indications of RCAVF. ESRD patients who had previously had an AVF, sepsis, hematological disorders, active tumoral status, a personal history of major surgery in the previous six months, or autoimmune diseases were all excluded. Furthermore, patients with no palpable thrill at the level of anastomosis immediately after AVF construction and patients with no sign of permeability at the level of AVF at 4 and 8 weeks of follow-up were also excluded.

The patients included were divided into two groups based on their AVF maturation status at 8 weeks: “Maturation” (Group 1) and “Failed Maturation” (Group 2).

### 2.2. Data Collection

The sex, age, and comorbidities of the patients were retrieved from the hospital’s computerized database. The same surgeon performed all RCAVFs and AVFs. An ultrasound assessment of the cephalic vein quality was performed initially, followed by a clinically perceptible pulse at the radial artery level. Regarding the laboratory parameters, we included in this study the laboratory data from samples taken pre-operatively. In terms of systemic inflammatory markers, we measured the IL-6 level and hematological ratios (neutrophil–lymphocyte ratios (NLR), platelet–lymphocyte ratios (PLR), and the systemic inflammatory index (SII)) [3,36,37,38,39,40,41,42,43,44,45,46,47,48,49,50,51,52]. The patients provided written informed consent for the publication of any data.

Inflammatory markers and Ca-P product were calculated according to the following formulas:

– NLR = the ratio between the total number of neutrophils and the total number of lymphocytes;
– PLR = the ratio between the total number of platelets and the total number of lymphocytes;
– SII = the total number of neutrophils multiplied by the total number of platelets, divided by the total number of lymphocytes;
– Ca-P product = calcium level multiplied by the phosphate level.

In terms of maturation requirements, we employed the “rule of 6” in our clinic, which is based on the following guidelines: a minimum flow of 600 mL/min, a vein diameter of 6 mm, a punctionable length of the venous segment more than 6 cm, and a maximum depth of 6 mm [1].

### 2.3. The Histopathology and Immunohistochemistry of Veins Obtained during the Creation of Arteriovenous Fistula

Samples of 5–10 mm length circumferential venous segments were collected during of surgical creation of AVF fixed in 10% neutral buffered formalin, sent for histological analyses, and embedded in paraffin. The histological features of tissue samples were examined in 4–5 µm hematoxylin and eosin (H&E)- and Elastic van Gieson-stained sections (Verhoeff Van Gieson/EVG Stain’ kit, Abcam, ab150667) by a senior pathologist (EH) who was blinded to the patient’s characteristics. The microscopic examination followed the most important vascular remodeling changes: focal or diffuse intimal hyperplasia, media hypertrophy, intima/media neovascularization, mononuclear inflammatory infiltrate around the vessels in the adventitia, disruption of the internal and external elastic lamina by loss of elastic fibers, foci of microcalcification, and the presence of intraluminal thrombi. The endothelial layers of neoformed vessels at the intima and media thickness were visualized via immunohistochemistry using anti-CD31 mouse monoclonal antibody; clone 1A10 (Bio SB, US). EnVision FLEX/HRP (Agilent, Dako) was used as secondary antibody in combination with 3,3′-diaminobenzidine chromogen (DAB) substrate, in order to give the reaction product a brown color. Nuclei were counterstained with hematoxylin. For the negative control, normal serum was substituted for the primary antibody.

### 2.4. Quantitative Digital Image Analysis: Digital Morphometry

**Figure 1:**
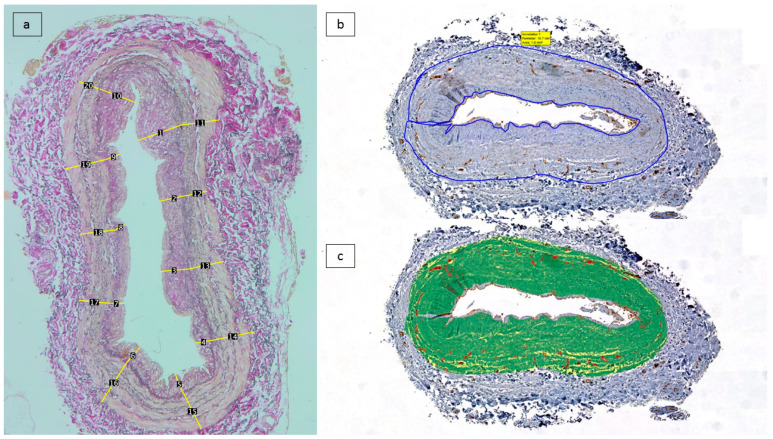
Scanned tissue areas corresponding to the intima and media were considered for analysis. (**a**) Morphometric analysis of average intimal and medial thickness by 10 measurements: Elastin van Gieson-stained sections). (**b**) Tissue segmentation on anti-CD31/DAB immunolabelled tissue sections, followed by annotation of region of interest (intima and media). (**c**) Application of trained algorithm on the region of interest measurment of the thresholded area.

(a) A morphometric analysis of intimal thickness was performed on Elastin van Gieson-stained slides. Microphotographs were taken using a microscope mounted to a Zeiss AxioCam digital camera from representative regions containing the whole wall thickness at 40× magnification. The resulting images were imported into ImageJ software (National Institute of Health, Bethesda, ML, USA). The internal elastic lamina (IEL) was traced, and on it, ten points with an equal distance from each other were considered. From these points, calibrated linear measurements were effectuated perpendicularly to the IEL, measuring the intimal thickness (from the IEL to the intimal surface) and the medial thickness (from the IEL to the external elastic lamina), respectively. The average of the ten measurements represented the average intimal and medial thickness (Figure 1a). The intimal media thickness (IMT) represents the sum of the thicknesses of the media and intima.

(b) A study of vascular density was carried out. Analysis of CD31 immunostained sections was performed at the 1st Department of Pathology and Experimental Cancer Research, Semmelweis University. The Panoramic Scan System (3D Histech Ltd., Budapest, Hungary) was used to digitize these 42 slides. Using the Panoramic Viewer program, tissue sections corresponding to the vascular well were manually annotated while excluding the endothelial lining of the intima and the vasa vasorum containing adventitia. Within the selected annotations, the Pattern Quant module of the Quant Center software package (3D Histech) was trained to provide automated differentiation between CD31-stained vascular structures, the background, and counterstained tissues (Figure 1b–c). The segmented areas were measured in µm^2^, and the positive surface area was estimated from the results.

### 2.5. Study Outcomes

The primary outcome was maturation failure at 8 weeks. The secondary endpoint was the existence of IH during morphometrical analysis, which was a shared endpoint of the presence of IH and maturation failure.

### 2.6. Statistical Analysis

SPSS for Mac OS version 28.0.1.0 (SPSS, Inc., Chicago, IL, USA) was used for statistical analysis. Chi-square tests were used to examine associations between inflammatory markers derived from total neutrophil, platelet, and lymphocyte counts, as well as IL-6, serum calcium, serum phosphate, Ca-P product, and intimal hyperplasia, while Student’s *t* tests or Mann-Whitney U tests were used to evaluate differences between continuous variables. To determine the association between systemic inflammatory markers and CD31-positive relative surfaces, we used the Spearman correlation. A multivariate logistic regression analysis of variables with *p* < 0.1 was used to identify independent predictors of vascular access maturation failure.

## 3. Results

During the study period, all of the included patients met all of the inclusion and exclusion criteria, with 12 patients (28.57%) having failed AVF maturation at 8 weeks. There was no difference in the demographic data. In terms of comorbidities, the second group had a greater incidence of heart failure (66.67% vs. 30%, *p* = 0.03), diabetes (91.67% vs. 53.33%, *p* = 0.04), peripheral artery disease (83.33% vs. 26.67%, *p* = 0.002), and obesity (83.33% vs. 40%, *p* = 0.01) (Table 1).

Additionally, regarding the laboratory findings, the patients with failure of maturation AVF had higher levels of serum uric acid (8.6 mg/dL vs. 6.09 mg/dL, *p* = 0.0005), serum phosphate (7.56 vs. 4.12, *p* < 0.0001), and creatinine (7.76 mg/dL vs. 5.92 mg/dL, *p* = 0.02), as well as lower levels of total calcium (1.9 mmol/L vs. 2.27 mmol/L, *p* = 0.0002), monocytes (0.66 vs. 0.84, *p* = 0.008), and lymphocytes (1.14 vs. 1.85, *p* < 0.0001). Moreover, all inflammatory markers (*p* = 0.001; *p* < 0.0001; *p* = 0.006, and *p* = 0.03) and Ca-P product (56.52 vs. 38.83, *p* < 0.0001) had higher baseline values in the patients with failure of AVF maturation. Upon immunohistochemical analysis, regarding the presence of CD31 immunolabelling, there was a higher incidence of CD31-positive surfaces (*p* = 0.006) and CD31-positive relative surfaces (*p* = 0.001) (Table 1).

**Figure 2:**
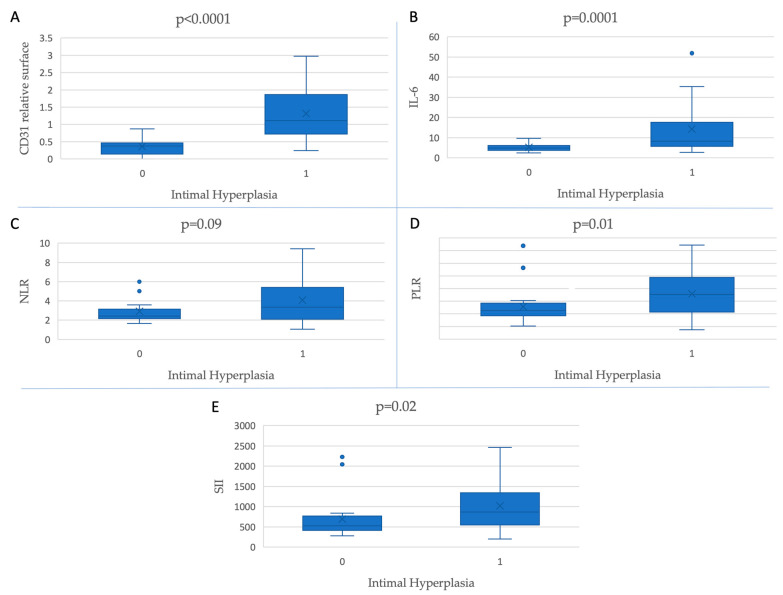
Inflammatory markers and CD31-positive relative surfaces’ association with the presence of intimal hyperplasia.

As shown in Figure 2, increased baseline levels of CD31-positive relative surfaces (*p* < 0.0001) and all inflammatory markers (PLR (*p* = 0.01), SII (*p* = 0.02), and IL-6 (*p* = 0.0001)) except for NLR (*p* = 0.09) are associated with IH.

**Figure 3:**
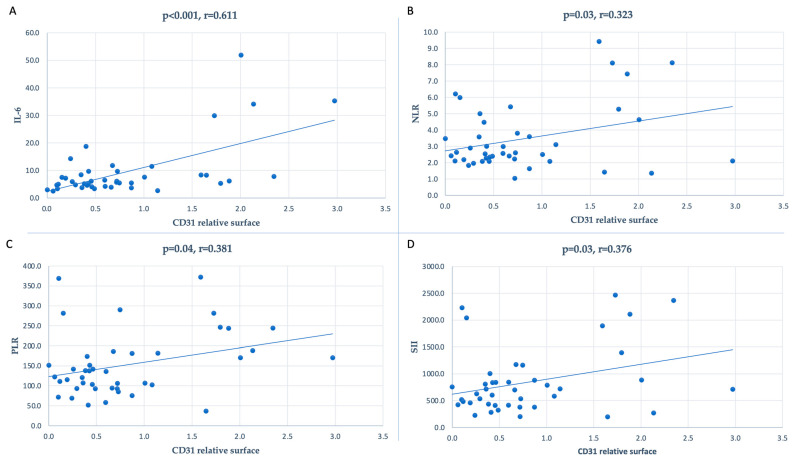
Correlation between the inflammatory markers and CD31-positive relative surfaces: (**A**) for IL-6 (r = 0.611, *p* < 0.001), (**B**) for NLR (r = 0.323, *p* = 0.03), (**C**) for PLR (r = 0.381, *p* = 0.04), and (**D**) for SII (r = 0.376, *p* = 0.03).

For a more in-depth analysis of the interrelationships between the systemic inflammatory status and CD31-positive surfaces in the thickened intima, we also investigated whether a higher value of inflammatory markers was correlated with a higher presence of CD31-positive microvessel density in the neointimal surface. We found that the NLR (r = 0.323; *p* = 0.03), PLR (r = 0.381; *p* = 0.04), SII (r = 0.376; *p* = 0.03), and IL-6 (r = 0.611; *p* < 0.001) were all positively significantly correlated with CD31 density (Figure 3).

A multivariate logistic regression analysis showed that the presence of heart failure (OR:3.71; *p* = 0.008), peripheral arterial disease (OR:3.82; *p* = 0.006), obesity (OR:2.28; *p* = 0.02), pre-operative intimal hyperplasia (OR:5.18; *p* = 0.03), CD31-positive relative surfaces (OR:10.65, *p* = 0.001), all high baseline values of systemic inflammatory markers (for all *p* < 0.05), high baseline values of serum phosphate (OR:7.43, *p* = 0.004), and Ca-P product (OR:4.51, *p* = 0.006) are predictors of vascular access maturation failure (Table 2). Additionally, the high baseline level of total serum calcium acts as protective factor against vascular access maturation failure (OR:0.17, *p* = 0.002) (Table 2).

## 4. Discussion

Arteriovenous fistula maturation, patency, and causes of non-maturation and dysfunction are the main topics discussed in the literature regarding end-stage kidney disease. The predictive value of systemic inflammatory markers in AVF dysfunction has been discussed in many papers, but the impact of CKD in intimal hyperplasia at the level of the venous component remains a complex mechanism not yet elucidated.

In our study, we used histopathological data as well as laboratory biomarkers to demonstrate the role of systemic inflammation in arteriovenous fistula dysfunction, the main finding being the correlation between systemic inflammatory markers and microvascular density in intimal hyperplasia. High values of inflammatory markers, the presence of IH, and intima neovascularization (as CD31-positive surface) are predictive factors of AVF dysfunction. Additionally, heart failure, peripheral arterial disease, and obesity are strong predictors of 8-week AVF maturation failure.

The association between vascular remodeling, intimal hyperplasia, and AVF maturation failure has been discussed in recent articles published in the literature, but with inconsistent results. Few studies have investigated the occurrence of neointimal hyperplasia at the venous component and its influence on maturation failure [26,34,53,54,55]. Duque et al. [53], who studied the impact of vasa vasorum density and vasa vasorum area in all layer vein samples taken at the time of AVF with maturation failure using anti-CD31 immunohistochemistry, found a correlation between lower vascularization of the media and an increase in postoperative intimal thickness (r = 0.53, *p* = 0.003 and r = 0.37, *p* = 0.045).

Based on comprehensive immunohistochemical research, Shehadeh et al. [54] demonstrated early remodeling 7 days after AVF creation, which needed ligation due to steal syndrome. Because of enhanced intramural oxygenation, the authors observed increased wall neovascularization and the presence of CD163+ macrophages. 

In terms of systemic inflammation, Pasqui et al. [56] discovered that high NLR levels (HR: 2.53, *p* = 0.01) and the presence of diabetes mellitus (HR: 1.41; *p* = 0.04) are independent predictors of a malfunctioning AVF in a study of 178 patients with AVF. Usman et al. [57] and Yilmaz et al. [58] found that high NLR levels are related to vascular access dysfunction and the occurrence of AVF stenosis. Sarioglu et al. [59] demonstrated that high PLR values correlate with AVF stenosis and thrombosis, and in the recent work published by our group of researchers [3], we demonstrated that high values of NLR, PLR, and SII, as well as the presence of heart failure (OR:4.38, *p* < 0.001) and diabetes mellitus (OR:5.63, *p* < 0.001) are predictors of AVF maturation failure in the case of 125 patients.

The serum levels of calcium, phosphate, and Ca-P product have an important role in the homeostasis and optimal functioning of the cardiovascular system [3,60,61]. According to the results of our study, the low level of serum calcium (*p* = 0.0002), the increased level of serum phosphate (*p* < 0.0001), and the increased values of Ca-P product (*p* < 0.0001) are associated with AVF maturation failure. Moreover, as can be seen in Table 2, a high baseline value of serum phosphate (OR:7.43, *p* = 0.004) and Ca-P product (OR:4.51, *p* = 0.006) are predictors of vascular access maturation failure. Additionally, the high baseline level of total serum calcium acts as protective factor against vascular access maturation failure (OR:0.17, *p* = 0.002). Similar to our results, Tuysuz et al. [60] demonstrated in a group of 79 patients that serum levels of phosphate (OR: 1.85, *p* = 0.05) and Ca-P product (OR: 1.11, *p* = 0.03) were predictive factors of AVF re-operation. Moreover, Unver et al. [61] demonstrated that high values of Ca-P product are associated with lower blood flow rates (*p* = 0.03). 

Additionally, the results of this study follow those of the previously published study by Kaller et al. [3], in which we demonstrated that high values of systemic inflammatory markers and Ca-P product are predictors of maturation failure (all *p* < 0.05).

The main reasons for AVF maturation failure and long-term AVF dysfunction are vascular remodeling and intimal hyperplasia [62,63]. Wong et al. [62] discovered that the density of CD31-positive cells increased, beginning on day 7, in a new mouse model of AVF failure. Furthermore, Cai et al. [63] discovered, in an animal model experimental investigation, that at day 14 post-operatively, there was a significant increase in the CD31 index in the angioplasty-treated vessel (*p* < 0.0001).

Among the study’s strengths are the quantitative measurement of intimal and media thickness and the vascular density (based on anti-CD31 immunolabelling), that is, the examination of inflammatory markers that play a role in the prediction of AVF maturation failure. This study, on the other hand, has a number of shortcomings. First and foremost, the small number of patients involved in this study originate from a single center. Secondly, because we quantitatively rather than qualitatively (immunohistochemically) assessed the local inflammatory infiltrate, we were unable to correlate these two markers of systemic inflammation. Also, due to the retrospective nature of the study, we do not have data on the patients’ chronic medications and cannot determine if these medications affect the inflammatory markers.

Questions regarding the correlation between the inflammatory markers and fistula dysfunction look like they are on their way to being clarified. Because of the low number of patients included in this study, further research must be conducted to demonstrate these results in a larger number of patients, so that they gain true value.

## 5. Conclusions

According to our findings, some comorbidities, including heart failure, peripheral artery disease, diabetes, and obesity, had a higher incidence in group 2 patients (the failed maturation group); regarding the laboratory findings, these patients had higher levels of serum uric acid and creatinine, as well as lower levels of total calcium, total bilirubin, monocytes, and lymphocytes.

The systemic inflammatory biomarkers (NLR, PLR, SII, and IL-6) showed an association with intimal hyperplasia. During morphometrical analysis, we demonstrated that CD31-positive relative surfaces have a higher baseline value in group 2 patients, and are also associated with intimal hyperplasia. The correlation test between a higher baseline value of the systemic inflammatory markers and a higher CD31-positive relative surface in the intimal hyperplasia area showed a positive result. 

Concluding all these findings, we can declare that the mentioned comorbidities, the systemic inflammatory markers (NLR, PLR, SII, IL-6), intimal hyperplasia, and CD31-positive relative surfaces are predictors of arteriovenous fistula maturation failure. By determining these parameters before planning the procedure, we can ensure preoperative risk group stratification and better patient management.

## Figures and Tables

**Table 1:** Demographic data, comorbidities, risk factors, morphometric and immunohistochemical analysis of all patients.

Variables		All Patients *n* = 42	Group 1 *n* = 30	Group 2 *n* = 12	*p* Value
Age mean ± SD (min–max)		67.07 ± 10.2 (39–86)	66.83 ± 10.58 (39–81)	67.66 ± 9.62 (54–86)	0.80
Male sex no. (%)		24 (57.14%)	18 (60%)	6 (50%)	0.55
Comorbidities and risk factors, no. (%)					
Arterial hypertension		31 (73.81%)	22 (73.33%)	9 (75%)	0.91
Atrial fibrillation		12 (28.57%)	7 (23.33%)	5 (41.67%)	0.24
Heart failure		17 (40.48%)	9 (30%)	8 (66.67%)	0.03
Diabetes mellitus		27 (64.29%)	16 (53.33%)	11 (91.67%)	0.04
History of stroke		6 (14.29%)	4 (13.33%)	2 (16.67%)	0.78
History of myocardial infarction		7 (16.67%)	3 (10%)	4 (33.33%)	0.08
Peripheral arterial disease		18 (42.86%)	8 (26.67%)	10 (83.33%)	0.002
Active smoking		27 (64.29%)	19 (63.33%)	8 (66.67%)	0.83
Obesity		22 (52.38%)	12 (40%)	10 (83.33%)	0.01
Laboratory data, median [Q1-Q3]					
Hemoglobin g/dL		10.05 [(8.22–11.07)	9.95 (8.22–10.96)	10.25 (8.95–11.22)	0.39
Hematocrit %		30.6 (25.8–34.92)	30.6 (25.57–34.85)	30.75 (29.02–34.8)	0.41
Glucose mg/dL		107.5 (92.4–142.7)	107.5 (89.6–136.5)	115.15 (96–155.4)	0.21
Serum uric acid mg/dL		6.5 (5.25–8.1)	6.09 (4.65–7.02)	8.6 (7.62–9.47)	0.0005
Total calcium mmol/L		2.2 (2.02–2.4)	2.27 (2.18–2.44)	1.9 (1.86–2.12)	0.0002
Total calcium mg/dL		8.79 (8.12–9.68)	9.07 (8.73–9.78)	7.59 (7.46–8.50)	0.0002
Serum phosphate mg/dL		4.74 (3.35–5.99)	4.13 (3.10–4.77)	7.20 (6.34–8.27)	<0.0001
Blood urea nitrogen mg/dL		131.15 (105.4–163.12)	130.15 (107.12–162.4)	144.9 (104.5–178.3)	0.30
Creatinine mg/dL		6.26 (4.97–7.7)	5.92 (4.8–6.88)	7.76 (6.38–8.46)	0.02
K mmol\L		5.25 (4.77–5.65)	5.09 (4.77–5.65)	5.44 (5.08–5.65)	0.17
Na mmol\L		138.5 (137–140)	139.5 (137–141)	137.5 (137–139.25)	0.09
International normalised ratio		1.07 (1.02–1.09)	1.07 (1.01–1.10)	1.06 (1.02–1.08)	0.29
Eosinophil × 10^3^/μL		0.16 (0.1–0.33)	0.19 (0.1–0.37)	0.12 (0.05–0.23)	0.08
Monocyte × 10^3^/μL		0.77 (0.65–0.91)	0.84 (0.67–0.96)	0.66 (0.55–0.72)	0.008
Lymphocytes × 10^3^/μL		1.72 (1.22–2.1)	1.85 (1.45–2.67)	1.14 (1.06–1.19)	<0.0001
Neutrophils × 10^3^/μL		5.39 (4.10–6.29)	5.19 (3.97–6.13)	5.85 (4.85–7.59)	0.08
PLT × 10^3^/μL		224.5 (193.25–281.75)	220.5 (185.25–270)	273.5 (200.25–312.15)	0.057
NLR		2.60 (2.13–4.31)	2.47 (2.09–3.00)	5.71 (4.03–7.61)	0.001
PLR		136.87 (96.76–181.78)	109.55 (93.08–140.97)	244.25 (182.37–281.56)	<0.0001
SII		706.5 (426–885.5)	561 (417.4–779.6)	1644.3 (843.1–2138.2)	0.0006
IL-6 pg/mL		5.82 (4.32–8.33)	5.32 (4.11–7.45)	7.66 (5.61–16.32)	0.03
Ca-P Product		43.47 (29.19–50.31)	38.83 (27.44–46.59)	56.52 (49.87–71.41)	<0.0001
Morphometric analysis, median [Q1–Q3]					
Intima Layer	Mean thickness	62.13 (15.98–110.79)	48.39 (15.98–96.96)	67.2 (20.9–174.3)	0.17
	Minimum thickness	8.36 (5.78–27.38)	7.40 (5.78–22.38)	22.72 (5.71–48.22)	0.18
	Maximum thickness	138.7 (38.19–282.9)	138.7 (36.8–260.1)	163.4 (68.7–347.9)	0.15
	Max/Min	9.43 (4.76–13.72)	8.71 (4.38–24.33)	9.79 (6.07–11.56)	0.41
Media Layer	Mean thickness	192.9 (138.3–294.9)	209.7 (141–292.5)	171.1 (111.6–302.9)	0.33
	Minimum thickness	128.04 (76.5–150.2)	129.3 (88.63–150.9)	107.6 (68.72–145.6)	0.22
	Maximum thickness	308.9 (194.6–453.5)	311.37 (201.8–453.5)	262.6 (179.3–421.1)	0.36
	Max/Min	2.48 (2.12–3.51)	2.43 (2.1–3.38)	2.65 (2.36–3.89)	0.16
IMT	Mean thickness	269.9 (173.2–391.9)	277.7 (181.2–366.4)	253.1 (161.2–472.6)	0.41
	Minimum thickness	162.1 (100.7–230.3)	168.1 (110.8–226.1)	137.5 (89.3–237.9)	0.32
	Maximum thickness	395.1 (243.3–593.2)	411.7 (262.8–535.6)	394.2 (236.1–658.6)	0.37
	Max/Min	2.5 (1.96–3.57)	2.25 (1.96–3.42)	3.04 (1.99–4)	0.22
Immunohistochemical analysis, median [Q1-Q3]					
CD 31, μm^2^		10,563.27 (1958.1–26,496.5)	4144.82 (1622.88–16,631.1)	35,706.1 (12,703.5–87,264.1)	0.006
CD31-POSITIVE relative surface, %		0.60 (0.35–1.06)	0.44 (0.30–0.72)	1.76 (0.70–2.03)	0.001

**Table 2:** Predictors of vascular access maturation failure.

	Vascular Access Maturation Failure		
	OR	95% CI	*p* Value
Heart failure	3.71	1.41–9.71	0.008
Diabetes mellitus	1.67	0.66–4.22	0.27
Peripheral arterial disease	3.82	1.43–10.21	0.006
History of myocardial infarction	1.45	0.54–3.61	0.48
Obesity	2.28	1.13–7.21	0.02
Intimal hyperplasia	5.18	1.15–23.29	0.03
CD31-positive relative surface	10.65	2.51–45.07	0.001
Total calcium mg/dl	0.17	0.05–0.52	0.002
Serum phosphate mg/dl	7.43	1.89–29.11	0.004
Ca-P product	4.51	1.41–18.28	0.006
NLR	2.61	1.43–4.78	0.002
PLR	1.02	1.01–1.04	0.02
SII	1.003	1.001–1.006	0.04
IL-6	1.15	1.01–1.30	0.03

## Data Availability

The data that support the findings of this study are available from the corresponding author upon reasonable request.
